# Using a higher criticism statistic to detect modest effects in a genome-wide study of rheumatoid arthritis

**DOI:** 10.1186/1753-6561-3-s7-s40

**Published:** 2009-12-15

**Authors:** Elena Parkhomenko, David Tritchler, Mathieu Lemire, Pingzhao Hu, Joseph Beyene

**Affiliations:** 1 Biostatistics Methodology Unit, Research Institute, Hospital for Sick Children, 555 University Avenue, Toronto, Ontario M5G 1X8, Canada; 2 Dalla Lana School of Public Health, University of Toronto, Health Sciences Building, 155 College Street, Toronto, Ontario M5T 3M7, Canada; 3 Department of Biostatistics, State University of New York at Buffalo, 249 Farber Hall, 3435 Main Street, Building 26, Buffalo, New York 14214-3000 USA; 4 Division of Epidemiology and Statistics, Ontario Cancer Institute, 610 University Ave, Toronto, Ontario M5G 2M9, Canada; 5 Ontario Institute for Cancer Research, MARS Centre, South Tower, 101 College Street, Suite 800, Toronto M5G 0A3, Canada; 6 Program in Genetics and Genomic Biology, The Hospital for Sick Children Research Institute, 15-706 TMDT, 101 College Street, Toronto, Ontario M5G 1L7, Canada

## Abstract

In high-dimensional studies such as genome-wide association studies, the correction for multiple testing in order to control total type I error results in decreased power to detect modest effects. We present a new analytical approach based on the higher criticism statistic that allows identification of the presence of modest effects. We apply our method to the genome-wide study of rheumatoid arthritis provided in the Genetic Analysis Workshop 16 Problem 1 data set. There is evidence for unknown bias in this study that could be explained by the presence of undetected modest effects. We compared the asymptotic and empirical thresholds for the higher criticism statistic. Using the asymptotic threshold we detected the presence of modest effects genome-wide. We also detected modest effects using 90^th ^percentile of the empirical null distribution as a threshold; however, there is no such evidence when the 95^th ^and 99^th ^percentiles were used. While the higher criticism method suggests that there is some evidence for modest effects, interpreting individual single-nucleotide polymorphisms with significant higher criticism statistics is of undermined value. The goal of higher criticism is to alert the researcher that genetic effects remain to be discovered and to promote the use of more targeted and powerful studies to detect the remaining effects.

## Background

Multiple genetic association studies of rheumatoid arthritis (RA) have reported inconsistent results [1]. It is hypothesized that these inconsistencies may be explained by inability to detect modest effects due to insufficient sample size [1]. In the case of high-dimensional studies such as genome-wide association studies, the correction for multiple testing in order to control total type I error results in decreased power to detect moderate effects. In a genome-wide study of RA conducted by [2], the authors report that after accounting for known significant single-nucleotide polymorphisms (SNPs) and possible population stratification, there is an inflation in the tail of the distribution of *p*-values that could indicate unknown bias in the study. Another explanation of this deviation from the expected distribution could be the presence of undetected, and therefore unexplained, modest effects. It has been reported that even for a larger data set that contains the provided data as a subset, the power to detect a disease-associated allele with population frequency of 0.2 and an odds ratio of 1.3 is only 13%, while for an odds ratio of 1.5 the power is 90% [2]. Thus, there is a limited ability to detect modest effects even with a larger sample size. We present a new approach that allows the determination of whether modest effects are present. Our technique is based on the higher criticism (HC) statistic of Donoho and Jin [3].

## Materials and methods

### Data

The data consist of 545,080 SNPs genotyped for 868 cases from the North American Rheumatoid Arthritis Consortium (NARAC) and 1194 controls. This is a subset of the Stage 1 data previously analyzed by Plenge et al. [2], after removing duplicated and contaminated samples. A detailed description of the complete data set and collection procedures can be found in Plenge et al. [2]. The data were offered as part of the Genetic Analysis Workshop 16.

We performed quality-control filtering of SNPs following the procedures in Plenge et al. [2]. We removed SNPs with more than 5% missingness, minor allele frequencies below 0.01, and based on Hardy-Weinberg equilibrium (*p *< 10^-5^). Because no information on the ancestry was provided, we assume that all related subjects and subjects with non-Europian ancestry were removed [2].

### Statistical Analysis

We applied the refined version of the HC statistic of Donoho and Jin [3] to test whether all remaining SNPs come from the null distribution and are not associated with RA versus an alternative hypothesis that there is a small number of moderate effects. The HC test can be treated as a test of mixing proportion in a mixture distribution with two components [3]: modest effects with probability density function (pdf) *f*_1 _and null effects have pdf *f*_0_. Then the pdf for the mixture distribution is *f *= *ε f*_1 _+ (1-*ε*)*f*_2 _The HC test for presence of modest effects is equivalent to testing *H*_0_: *ε *= 0.

The HC test utilizes individual *p*-values and is implemented as follows

[3,4]. Let *p*_(1) _<*p*_(2) _<...<*p*_(*n*) _be individual SNP test *p*-values sorted in ascending order. Also let

Then the HC statistic is

for *α *= 0.05 level test. We reject the null hypothesis that there are no significant effects when 

We obtained a *p*-value for each SNP that passed quality-control filtering using the test of genetic association implemented in PLINK [5]. According to Plenge et al. [2] there is evidence of population substructure in the given sample, with chi-square statistics inflated by a factor of 1.43. We adopted the approach of Plenge et al. [2] based on principal components to account for population stratification. We used the eigenvectors of a covariance matrix between all DNA samples as surrogates for ancestry [6]. Approximately 120,000 autosomal SNPs with pairwise correlation less than 0.3 were used to calculate the covariance matrix. Following Plenge et al. [2], we did not include SNPs on the short arms of chromosomes 6 and 8 in this calculation. We recomputed the eigenvectors after removing seven outliers identified by inspecting the eigenvectors associated with the top ten eigenvalues. As in Plenge et al. [2], we chose the top three vectors that were statistically significant predictors of case-control status to correct for population stratification and included them as covariates in a logistic regression model in PLINK. We obtained the inflation factor of all association results, excluding results on the short arm of chromosome 6 (*λ*_*GC *_= 1.035), which is similar to one in Plenge et al. [2].

The HC test evaluates evidence of modest effects that could be present in the data in addition to the significant effects already identified. Therefore, we applied the HC test genome-wide after removing known significant effects, which were defined as regions identified in the previous studies as associated with RA on a genome-wide level. Excluded SNPs were from the extended MHC region [1] from HIST1H2AA to K1FC1, the TRAF1-C5 region [2] extended to PHF19-C5 because of linkage disequilibrium, and the PTPN22 region [1]. The base-pair positions for excluded regions were identified using hg16 map provided with the data. We recomputed the inflation factor for the remaining SNPs and obtained  = 1.030. We obtained *p*-values corresponding to chi-square statistics from the logistic regression model described above; these were also corrected for the residual inflation by dividing by .

We compared the use of the asymptotic threshold for the HC statistic as in Cayon et al. [4], i.e., , to the empirical threshold. One thousand data sets from the null distribution were generated by permuting case/control status while keeping other variables constant. We applied the same logistic model with principal components computed for the original data as covariates and the same excluded SNPs as in the analysis of non-permuted data. We considered three options for the empirical threshold: 90^th^, 95^th^, and 99^th ^percentile of .

## Results

### Genome-wide analysis

**Figure 1:**
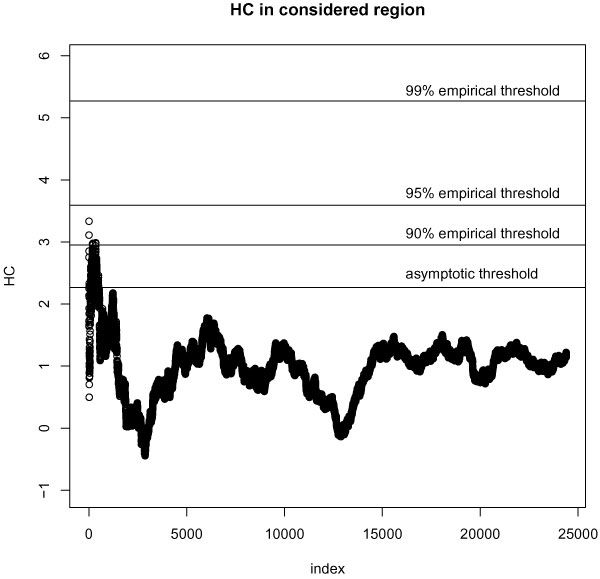
**Higher criticism statistics *HC*_*n*, *i *_for 24,402 SNPs**. Horizontal lines from bottom to top: asymptotic threshold, 90% empirical threshold, 95% empirical threshold, 99% empirical threshold.

There were 488,126 SNPs remaining after quality control filtering and removing regions with previously identified significant effects. Maximum HC was computed for 24,402 SNPs that satisfied 1 ≤ *i *≤ 0.05*n*, and *p*_(*i*) _≥ 1/*n *conditions and was equal to 3.333 while the genome-wide asymptotic threshold was 2.268. Figure 1 shows *HC*_*n*, *i *_statistics for the region over which maximum HC was computed. It also shows the asymptotic threshold. These results indicate presence of modest effects on a genome-wide level. Thus, after SNPs with known significant effects have been removed there is still evidence for association with RA that has not been explained. The highest *p*-value for which HC exceeds the threshold is 9.19 × 10^-4^, while the maximum of *HC*_*n*, *i *_corresponds to *p *= 7.46 × 10^-6^. There were 282 *HC*_*n*, *i *_statistics exceeding the asymptotic threshold, indicating modest effects.

### Empirical threshold

The values of 90^th^, 95^th^, and 99^th ^percentiles of  are 2.953, 3.591, and 5.259, respectively. Jin [4] suggested calling the HC statistic significant if it exceeds 99% of generated HC statistics from the null distribution. However, inspection of  statistics suggests that this empirical threshold could be driven by a small number of outliers. Therefore, the 90^th ^and 95^th ^percentiles of  could be more appropriate choices. When 90^th ^percentile is selected as threshold, seven HC statistics exceed the threshold, indicating the presence of modest effects. The *p*-values corresponding to these statistics range between 6.81 × 10^-6 ^and 6.21 × 10^-4^. There is no evidence of modest effects at the 95^th ^and 99^th ^percentile levels.

## Discussion

The HC statistic using the asymptotic threshold indicates the presence of modest effects on a genome-wide level. However, this threshold is not a boundary for a significance test, but rather a large-sample analytical result that applies to any data set, and gives the expectation of the HC statistic under the null hypothesis. It gives a crude idea of what values of HC start to be interesting, and displays the effect of the number of tests. Using an asymptotic threshold in this application may not be appropriate due to dependency between the individuals. Asymptotic assumptions discussed elsewhere [3,4] are not met in this study. We considered three options for the empirical threshold; for one of which provides evidence of modest effects, but not the other two. Thus, the choice of a percentile of null distribution to be used as a threshold has a direct effect on the conclusion about the presence of modest effects. Additional study of an appropriate empirical null distribution and empirical threshold is required.

The HC graph in Figure 1 demonstrates that there is no direct correspondence between *HC*_*n*, *i *_statistics and *p*-values in a sense that smaller *p*-values produce higher *HC*_*n*, *I *_values. In fact, the maximum of *HC*_*n*, *i *_corresponds to *p *= 7.46 × 10^-6^, which is not significant after correction for multiple testing using Benjamini-Hochberg rule [7]: *p*_*BH *_= 0.364. Thus, this effect is not significant enough to be detected by traditional approaches, which supports the usefulness of the HC statistic for detection of the presence of modest effects in the context of multiple hypothesis testing.

In addition, although the region where *HC*_*n*, *i *_statistics exceed the asymptotic threshold does not include the statistics corresponding to the smallest *p*-values, this is the region of interest. The region of *HC*_*n*, *i *_statistics above the threshold can be used to identify the range of *p*-values that could contain modest effects because it contains larger frequency of *p*-values in a specific range than expected by chance [4]. On the other hand, the region to the left that contains smaller *p*-values does not have a higher frequency of *p*-values than expected by chance. Therefore, the hypothesis is that the unidentified modest effects could be found in the range of *p*-values for which the *HC*_*n*, *i *_statistics exceed the threshold, while the conventional approach of considering only most extreme *p*-values up to a certain threshold may lead to missing modest effects. HC results could be used to alert a researcher that there is another range of larger *p*-values and smaller effect sizes that could be of interest and to promote the use of more targeted and powerful studies to detect the remaining genetic effects. Because the HC test is a global test of the presence of modest effects, caution should be exercised when trying to interpret individual SNPs with *p*-values in the range of interest.

## List of abbreviations used

HC: Higher criticism; NARAC: North American Rheumatoid Arthritis Consortium; PDF: Probability density function; RA: Rheumatoid arthritis; SNP: Single-nucleotide polymorphism.

## Competing interests

The authors declare that they have no competing interests.

## Authors' contributions

EP performed the statistical analysis and drafted the manuscript. All authors participated in the statistical analysis and helped to draft the manuscript. All authors read and approved the final manuscript.
